# Cardiovascular effects of relaxin-2: therapeutic potential and future perspectives

**DOI:** 10.1007/s00392-023-02305-1

**Published:** 2023-09-18

**Authors:** Nísia Almeida-Pinto, Thomas Bernd Dschietzig, Carmen Brás-Silva, Rui Adão

**Affiliations:** 1 Cardiovascular R&D Centre—UnIC@RISE, Department of Surgery and Physiology, Faculty of Medicine, University of Porto, 4200-319 Porto, Portugal; 2 Relaxera GmbH & Co. KG, Bensheim, Germany; 3 Faculty of Nutrition and Food Sciences, University of Porto, Porto, Portugal; 4 Department of Pharmacology and Toxicology, School of Medicine, Universidad Complutense de Madrid, Madrid, Spain; 5 CIBER Enfermedades Respiratorias (Ciberes), Madrid, Spain

**Keywords:** Relaxin, RXFP1 mimetics, Serelaxin, Cardiovascular diseases, Heart failure

## Abstract

The hormone relaxin-2 has emerged as a promising player in regulating the physiology of the cardiovascular system. Through binding to the relaxin family peptide receptor 1 (RXFP1), this hormone elicits multiple physiological responses including vasodilation induction, reduction of inflammation and oxidative stress, and angiogenesis stimulation. The role of relaxin-2, or its recombinant human form known as serelaxin, has been investigated in preclinical and clinical studies as a potential therapy for cardiovascular diseases, especially heart failure, whose current therapy is still unoptimized. However, evidence from past clinical trials has been inconsistent and further research is needed to fully understand the potential applications of relaxin-2. This review provides an overview of serelaxin use in clinical trials and discusses future directions in the development of relaxin-2 mimetics, which may offer new therapeutic options for patients with heart failure.

## Introduction

Relaxin-2 is an insulin-like peptide hormone, primarily secreted by the corpus luteum in response to the physiological changes associated with pregnancy [1]. It is widely recognized for its important role in maternity by allowing adaptations during pregnancy such as the pubic ligament relaxing and myometrium softening [2]. Despite its central role in pregnant women, this hormone has pleiotropic effects that are not exclusive to this population. Indeed, the actions of relaxin-2 have also been observed in males and non-pregnant women [3, 4].

Relaxin-2 can induce hemodynamic alterations, such as increasing cardiac output, arterial compliance, renal blood flow and glomerular filtration, as well as decreasing peripheral arterial resistance [5, 6]. Additionally, relaxin has a vasodilatory effect, stimulates angiogenesis, and prevents inflammation in the vasculature [5]. Pre-clinical studies have also shown that it can exhibit anti-fibrotic properties that may help to reduce fibrosis in organs like the liver, lungs, kidney and heart [7–9]. Given the evidence of multiple cardioprotective effects against major disease stigmas, clinical studies were initiated to discern whether exogenous relaxin administration can improve therapeutic approaches in cardiovascular diseases and overall patient outcomes [10]. In this article, we aim to review the clinical trials data involving serelaxin and explore its potential as a therapeutic agent. Additionally, we aim to identify potential gaps in our current understanding of serelaxin clinical effects and provide future directions which may help lead future research.

## Methods

We conducted a comprehensive literature search using online databases, including PubMed/MEDLINE, ScienceDirect, Scopus and ResearchGate. The search strategy included a combination of search terms: “relaxin”, “serelaxin”, “clinical trials”, “cardiovascular disease”, “heart failure”, “coronary artery disease”, “relaxin analogue”, “relaxin-2 mimetics” and “RXFP1 agonist”.

Also, we searched on ClinicalTrials.gov to identify clinical trials with serelaxin using the following search terms: “relaxin” “serelaxin” “cardiovascular diseases” “heart failure”.

Our methodology aimed to provide an overview of the current state of knowledge on relaxin and its clinical applications.

### Mechanisms of action of relaxin

**Fig. 1:**
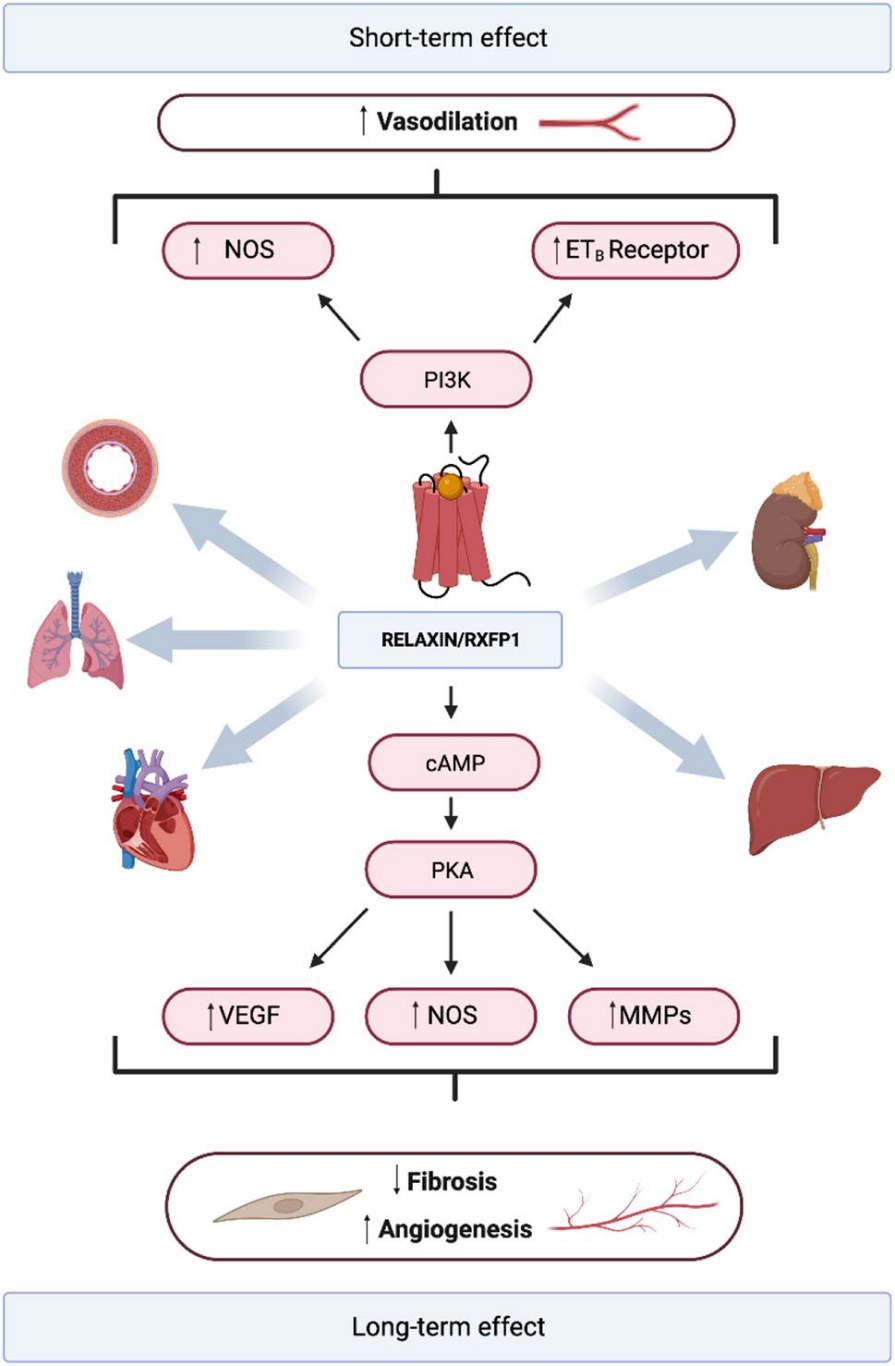
Relaxin’s actions are mediated by the RXFP1 receptor. Short-term exposure to relaxin primarily induces vasodilation by phosphorylating NOS (nitric oxide synthase) and enhancing the expression of the endothelial-B receptor (ET-B receptor). With sustained exposure, relaxin promotes an upregulation in gene expression of crucial factors such as vascular endothelial growth factor (VEGF), inducible NOS (iNOS), and matrix metalloproteinases (MMPs). This response culminates in a potent anti-fibrotic effect and in the promotion of angiogenesis. The cardioprotective impact of relaxin is reached through two main mechanisms: hemodynamic improvement and end-organ protection. These positive effects extend to the liver, kidneys, heart, lungs, and vasculature. *Created* with *BioRender*.com

Relaxin-2 pleiotropic effects are primarily mediated through binding to the relaxin family peptide receptor 1 (RXFP1) [11]. This, in turn, triggers the activation of different cascade pathways that mediate the hormone effects. The effects are produced mainly by the increase in nitric oxide (NO), cyclic AMP (cAMP), mitogen-activated protein kinases (MAPKs), matrix metalloproteinases (MMPs), angiogenic growth factors like vascular endothelial growth factor (VEGF) and upregulating the expression of endothelial-B receptor (ET-B) [12]. Moreover, the varying duration of relaxin exposure elicits distinct pathways. The relaxin acute effects (which occur within minutes) are mediated by the phosphoinositide-3-kinase (PI3K) pathway and involves the phosphorylation of NO synthase (NOS), which mainly leads to vasodilation [13]. In contrast, sustained relaxin exposure leads to changes in gene expression, increased VEGF, inducible NOS (iNOS) and MMPs expression, ultimately leading to its anti-fibrotic actions [13, 14] (Fig. 1).

## Results

### Heart failure

Over the past decades, the heart failure (HF) burden has become increasingly noticeable, as it represents the leading cause of hospitalization in patients older than 65 years of age [15]. Despite efforts to mitigate its impact on patient health, HF remains a significant clinical challenge, with a rising incidence and alarming rates of in-hospital mortality and readmissions [16]. The current guidelines recommended for acute heart failure (AHF) treatment still have some limitations such as the lack of consensus on the early management of HF decompensation. This may impede progress towards achieving better long-term outcomes [17]. Thus, new therapies are currently under research to improve patient long-term outcomes and reduce readmission rates [18].

Dschietzig et al. provided the first evidence of relaxin’s role in HF. The study revealed that endogenous relaxin, is not only constitutively expressed in humans but also exhibits varying levels according to the severity of congestive heart failure (CHF) [19]. Indeed, relaxin levels were significantly elevated in CHF patients and responded to the acute hemodynamic improvement during AHF therapy. Furthermore, the study showed that endogenous relaxin exerts a compensatory effect in CHF such as inducing vasodilation, diuresis, atrial natriuretic peptide release and suppression of the ET-1 system. These findings suggested that endogenous relaxin may play a role in the pathophysiology of the failing human heart and could be a promising target for future therapeutic interventions, as well as a potential marker for assessing prognosis [19]. Moreover, in a pilot clinical trial, Dschietzig et al. further demonstrated the systemic vasodilatory effect of intravenous human relaxin in patients with CHF. The study showed that treatment with intravenous human relaxin increased stroke volume and decreased pulmonary capillary wedge pressure (PCWP) without reducing blood pressure (BP) [20]. This finding inspired subsequent clinical trials to further explore the potential of recombinant human relaxin as a potential therapy for AHF management.

### Phase II

#### Pre-RELAX-AHF

The Pre-RELAX-AHF trial studied AHF patients to evaluate serelaxin dose–response effect on clinical outcomes, symptom improvement and safety [21]. The main results of this trial are summarized in Table 1. Serelaxin treatment unveiled diverse clinically significant outcomes in patients admitted for AHF with signs of congestion, normal-to-increased blood pressure, and mild-to-moderate renal dysfunction. Specifically, within 6 h of treatment, patients experienced a statistically significant improvement in dyspnea when compared to placebo, with a reported 17% absolute increase in the number of patients with symptom relief which was sustained for up to 14 days. Additionally, serelaxin-treated patients had a higher attenuation of congestion signs (oedema, rales, jugular venous pressure) compared to placebo, at days 5 and 14, as well as decreased worsening heart failure (WHF) at day 5 [22]. Moreover, relaxin-treated patients showed improvement in long-term outcomes, with an estimated 87% decrease in the risk of cardiovascular death (CVD) or re-hospitalization due to HF or renal failure at day 60, as well as improved 180-day survival (Table 1). During the trial, doses of 10 μg/kg, 30 μg/kg, 100, and 250 μg/kg per day were tested, concluding that the most beneficial dose was 48-h intravenous serelaxin at 30 μg/kg per day. This dose was chosen for further assessment in the subsequent phase III studies of serelaxin for AHF [21].

**Table 1:** Major phase 2 clinical trials on serelaxin

Clinical trial (Acronym)	Clinical trials. Gov identifier	Participants	CV condition	Intervention	Study duration	Main results
A multicenter, double-blind, randomized, parallel group, placebo-controlled study to evaluate the effects of intravenous serelaxin infusion on micro and macrovascular function in patients with coronary artery disease [38]	NCT01979614	- 58 Follow-up: 6 months Inclusion criteria: - > 18 years with proven obstructive CAD Exclusion criteria - AHF at baseline - HF NYHA Class III–IV	CAD	Treatment group Serelaxin: 30 μg/kg/day Control group Placebo	November 2013 to August 2016	- Significant BP reductions at 2 h (P = 0.0003) and 6 h (P = 0.001) - No significant change in MBF at rest (P = 0.40) or during stress (P = 0.76) - No significant change at 47 h in global MPR, in Alx or in aortic stiffness (P > 0.05 for all) - No significant change in PWV up to day 180 (P > 0.05 for all) - Significant decrease in cystatin C and endothelin-1 - No statistically significant changes in ventricular volumes and ejection fractions - Similar numbers of SAEs between treatment groups
Relaxin for the treatment of patients with acute heart failure: a multicentre, randomized, placebo-controlled, parallel-group, dose-finding phase IIb study (Pre-RELAX-AHF) [21]	NCT00520806	- 234 Follow-up: 6 months Time to randomization ± SD (hours): 8.4 ± 5.35 Inclusion criteria: - Within 16 h of presentation for AHF - SBP > 125 mm Hg - eGFR of 30–75 mL/min per 1.3 m^2^	AHF	Group 1 Relaxin:10 μg/kg/day for 48 h Group 2 Relaxin: 30 μg/kg/day for 48 h Group 3 Relaxin:100 μg/kg/day for 48 h Group 4 Relaxin 250: μg/kg/day for 48 h Control group Placebo for 48 h	December 2007 to August 2008	- Significant improvement in dyspnea at 24 h (P = 0.044) and up to day 14 (P = 0.053) - Significant reduction of CV death or readmission at 60 days (P = 0.053) - Decreased death due to HF or renal failure at day 60 (relaxin 6.1% vs placebo 17.2%) - Decreased all-cause mortality (P = 0.17) and CV mortality (P = 0.14) at day 180 - Decrease WHF through day 5 (P = 0.29) - Increased number of days alive out of the hospital by day 60 (P = 0.16) and decreased LoS (P = 0.18) - Similar number of AEs between groups
Pharmacokinetics & safety of serelaxin on top of standard of care therapy in pediatric patients with acute heart failure (RELAX-PEDS-PK) [23]	NCT02151383	- 12 Group 1 - 6 to < 18 years Group 2 - 1 to < 6 years Group 3 - 1 month to < 1 year Inclusion criteria: Hospitalized with AHF	AHF	Treatment - Low dose for 48 h 3, 10, 30 ug/kg/Day or - High dose for 48 h 10,30, 100 ug/kg/Day	May 2014 to April 2017	- Results not analyzed due to early termination - Number of patients with treatment-emergent AEs, SAEs, and death - Pharmacokinetic concentrations for low dose and high dose intervention
Study of safety, tolerability and pharmacokinetics of serelaxin in Japanese Acute Heart Failure (AHF) patients (RELAX-AHF-JAPAN) [56]	NCT02002702	- 46 Follow-up: 60 days Time to randomization (hours): 8.2 Mean age ± SD (years) Group 1: 70.2 ± 11.8 Group 2: 79.7 ± 9.0 Control: 76.4 ± 12.1	AHF	Group 1: 10 µg/kg/day serelaxin 48 h Group 2: 30 µg/kg/day serelaxin 48 h Control group: Placebo	August 2014	- Greater reduction in SBP with 10 µg/kg/day serelaxin compared to placebo - Greater reductions in NT-proBNP in 10 and 30 µg/kg/day serelaxin at day 2 and day 5 compared to placebo - AEs profile comparable between treatment groups
Safety of repeat doses of IV serelaxin in subjects with chronic heart failure (RELAX-REPEAT) [51, 57]	NCT01982292	- 321 Follow-up: 16 weeks Inclusion criteria: compensated CHF (NYHA Class II–III)	CHF	Treatment group Serelaxin: 30 μg/kg/day Control group Placebo	November 2013 to September 2015	- Non-statistically significant increase in the proportion of patient’s antibody positive - No confirmed hypersensitivity or infusion-related reactions in any treatment group - Statistically significant decreases in cystatin-C - Statistically significant increases in eGFR - Improved renal function following 48 h serelaxin - AEs profile comparable between groups
Study of the Effect of Serelaxin on High-sensitivity Cardiac Troponin I (Hs-cTnI) Release in Patients With Chronic Heart Failure (RELAX-CARDIO) [58]	NCT02625922	- 26 Mean age ± SD (years) Serelaxin -Placebo group: 74.7 ± 7.62 Placebo -Serelaxin group: 66.5 ± 13.89	CHF	IV Serelaxin (weight-range adjusted dosing regimen) IV Matching placebo (adjusted dosing regimen) Serelaxin -placebo treatment group: Subjects received serelaxin in treatment period 1 and placebo in treatment period 2 Placebo -Serelaxin Treatment group: Subjects received placebo in treatment period 1 and serelaxin in treatment period 2	December 2015 to January 2017	- Geometric mean of Hs-cTnI concentration after exercise compared to placebo The study was terminated early by Novartis, 19-Apr-2017 - hs-cTnI assay was not completed, and the primary efficacy endpoint was not analyzed

It includes the information on target disease, patient population, intervention dosage and key findings

*IV* intravenous, *SD* standard deviation, *CV* cardiovascular, *CAD* coronary artery disease, *AHF* acute heart failure, *NYHA* New York heart association, *HF* heart failure, *LoS* length of stay, *WHF* worsening heart failure, *CHF* congestive heart failure, *eGFR* estimated glomerular filtration rate, *AEs* adverse events, *SAEs* serious adverse events, *BP* blood pressure, *SBP* systolic blood pressure, *MPR* myocardial perfusion rate, *MBF* myocardial blood flow, *PWV* pulse wave velocity, *Alx* augmentation index, *Hs-cTnI* high-sensitivity cardiac troponin I, *NT-proBNP* N-terminal (NT)-pro hormone BNP

#### RELAX-PEDS-PK

Given the previous clinical results showing improvements in symptomatic management and long-term mortality in adults, researchers tried to test the safety and efficacy of serelaxin, in addition to standard of care (SoC) therapy, in children under 18 years hospitalized for AHF. However, this study was terminated early due to the RELAX-AHF-2 results, thus, the data were not statistically analyzed [23].

### Phase III

#### RELAX-AHF

A phase III study was conducted with the same inclusion criteria as Pre-RELAX-AHF, aiming to investigate whether patients treated with serelaxin would experience greater dyspnea relief than patients treated with SoC and placebo [24]. This study used two primary endpoints to define dyspnea improvement: the change in patient-reported dyspnea measured by the area under the curve (AUC) of the 100-point visual analogue scale (VAS) from baseline to day 5, and patient-reported dyspnea relative to the start of the study using the 7-level Likert scale in the first 24 h. The main results can be seen in Table 2. Compared to the placebo group, serelaxin-treated patients showed significant improvement in the first primary dyspnea endpoint measured by the VAS AUC. This improvement met the efficacy criteria of one primary endpoint with a p-value of < 0.025. However, there was no significant change in the patient-reported dyspnea endpoint. Despite this, serelaxin-treated patients reported a quicker dyspnea improvement than those in the placebo group (P = 0.002) [24]. Serelaxin showed no significant effect on CVD or readmissions and days alive out of the hospital up to day 60. Surprisingly, the study found a significant 37% reduction in both cardiovascular and all-cause mortality at day 180, as well as a 30% hazard reduction of WHF in the first 14 days (P = 0.02) [25]. Regarding the safety outcomes, serelaxin administration was well tolerated and no major adverse events (AEs) were reported. Also, patients treated with serelaxin had lower incidence of renal AEs [26]. However, since RELAX-AHF was not statistically powered to provide conclusive results on mortality, regulatory authorities requested a larger, sufficiently powered phase-III trial for approval.

**Table 2:** Major phase 3 clinical trials on serelaxin

Clinical trial (Acronym)	Clinical trials. Gov identifier	Participants	CV condition	Intervention	Study duration	Main results
Serelaxin, recombinant human relaxin-2, for treatment of acute heart failure: a randomized, placebo-controlled trial (RELAX-AHF) [24]	NCT00520806	1161 participants Follow-up: 180 days Time to randomization ± SD (hours): 7.9 ± 4.63 Mean age ± SD (years) Treatment group: 71.6 ± 11.7 Control group: 72.5 ± 10.8	AHF	Treatment group Serelaxin:30 μg/kg/day Control group Placebo	October 2009 to September 2012	- Significant improvement in dyspnea measured by VAS AUC (P = 0.007) - No significant improvement in patient-reported dyspnea (P = 0.70) - Significant reduction of WHF up to day 14 (P = 0.024) - Significant reduction of all-cause mortality (P = 0.019) and CV death (P = 0.028) at day 180 - Significant reduction of LoS by 0.9 days (P = 0.04) - No significant reduction of CV death or readmissions to day 60 (P = 0.89) - Significant decrease in AEs related to renal impairment (P = 0.03)
A multicenter, randomised, double-blind, placebo-controlled phase III study to evaluate the efficacy, safety and tolerability of serelaxin when added to standard therapy in acute heart failure patients (RELAX-AHF-2) [27]	NCT01870778	6545 participants Follow-up: 180 days Time to randomization ± SD (hours): 8.13 ± 4.49 Mean age ± SD (years): Treatment group: 73.1 ± 11.2 Control group: 72.8 ± 11.2	AHF	Treatment group Serelaxin 30 μg/kg/day Control group Placebo	June 2013 to February 2017	- No significant reduction of CV death at day 180 (P = 0.77) - No significant reduction of WHF at day 5 (P = 0.19) - Similar all-cause death and CV death or readmission 180 at days - Similar LoS - Similar number of AEs and SAEs
Efficacy, Safety and Tolerability of Sexelaxin When Added to Standard Therapy in AHF (RELAX-AHF- ASIA) [29, 32]	NCT02007720	876 participants Follow-up: 180 days Mean age ± SD (years): Treatment group: 68.9 ± 14.40 Control group: 70.2 ± 13.86	AHF	Treatment group Serelaxin 30 μg/kg/day Control group Placebo	December 2013 to June 2017	- No significant reduction in patients with treatment failure in the serelaxin group (4.1%) vs placebo group (8.3%) - Significant reduction of WHF at day 5 (HR = of 0.41, P = 0.0119) - No significant reduction in CV death and all-cause mortality at day 180 - Similar frequency of SAEs
Efficacy and safety of serelaxin when added to standard of care in patients with acute heart failure: results from a PROBE study (RELAX-AHF- EU)[33]	NCT02064868	2650 participants Follow-up: 30 days Mean age ± SD (years): Treatment group: 75.24 ± 10.349 Control group: 75.95 ± 9.905	AHF	Treatment group SoC + Serelaxin 30 μg/kg/day Control group SoC	February 2014 to April 2017	- Significant reduction of adjudicated WHF and all-cause of death through Day 5 (HR = 0.71 [95% CI 0.51–0.98] P = 0.0172) - No significant reduction of investigator-reported WHF or all-cause of death through day 5 (HR = 0.78 [95% CI 0.59–1.0] P = 0.029) - Significant reduction in persistent HF signs/symptoms up to day 4 (all P ≤ 0.01) - No significant reduction in WHF, all-cause death or HF readmissions through day 14 (P = 0.0634) - No significant change in LoS - Significant reduction in renal deterioration through day 5 and at discharge (all P ≤ 0.01) - AEs profile was similar

It includes the information on target disease, patient population, intervention dosage and key findings

*CI* confidence interval, *SD* standard deviation, *HR* hazard ratio, *CV* cardiovascular, *VAS* visual analogue scale, *AUC* area under the curve, *AHF* acute heart failure, *HF* heart failure, *LoS* length of stay, *WHF* worsening heart failure, *SoC* standard of care, *AEs* adverse events, *SAEs* serious adverse event

#### RELAX-AHF-2

The RELAX-AHF-2 trial was designed to investigate if serelaxin could reduce cardiovascular mortality and improve long-term patient outcomes through an intention-to-treat analysis [27]. The inclusion and exclusion criteria remained similar to the previous trials mentioned and have been described elsewhere [28]. The two primary endpoints were CVD at 180 days and WHF at day 5. The trial failed to meet both endpoints as the treatment with serelaxin did not show a statistically significant decrease in the incidence of CVD at day 180 nor WHF at day 5, as shown in Table 2. Moreover, the study found that both groups had similar rates of all-cause mortality, death from cardiovascular causes and readmission for heart failure or renal failure at day 180. However, these findings were not analyzed for significance. In this trial, serelaxin administration was still demonstrated to be safe as both groups experienced a comparable number of AEs [27].

#### RELAX-AHF-ASIA

RELAX-AHF-ASIA measured the impact of serelaxin on clinical outcomes and symptom alleviation in Asian patients with AHF [29]. The trial was expected to randomize 1520 patients, however, only 876 patients were randomized as the study was terminated prematurely due to RELAX-AHF-2 results. The study enrollment criteria were identical to RELAX-AHF and RELAX-AHF-2, however, this trial was designed with a novel endpoint [30]. Indeed, a composite endpoint was applied in this trial, consisting of 3 outcomes: (1) improvement in dyspnea (using patient-reported Likert Scale) and in signs of congestion, assessed by at least two physicians on Day 2, categorized as treatment success; (2) occurring of WHF demanding additional interventions, readmission owing to HF or renal failure or death up to day 5, representing treatment failure and (3) the patient does not meet the criteria for the treatment success nor the treatment failure, being labelled as unchanged. WHF up to day 5, cardiovascular and all-cause death at day 180 were also evaluated as secondary endpoints. In this trial, serelaxin was well tolerated and reduced the percentage of patients with treatment failure, although this was not statistically significant [31]. As seen in Table 2, the cardiovascular mortality and all-cause mortality through day 180 were similar in both serelaxin and placebo patients, despite the significant reduction in WHF at day 5 seen in serelaxin-treated patients. [29, 32].

#### RELAX-AHF-EUROPE

The RELAX-AHF-EU assessed the impact of serelaxin in combination with SoC therapy on the proportion of patients presenting with WHF or death from all causes up to day 5, in hospitalized patients admitted for AHF across Europe [33]. The patients included in this study remained similar to the ones included in the previously discussed trials. This study followed a prospective, randomized, open-label, blinded-endpoint validation design (PROBE). Out of 3183 patients targeted, only 2666 were randomized (2:1). Patients received either the Soc therapy alone (consisting of oxygen, loop diuretics, vasodilators and inotropes) or Soc therapy along with the continuous intravenous infusion of serelaxin for 48 h [33]. The main results can be found in Table 2. This study showed an overall significant reduction of 1.9% in the risk of adjudicated WHF or all-cause mortality through day 5 in the group of patients that received serelaxin treatment additionally to SoC therapy. However, when considering all events reported by investigators (independently of the Clinical Endpoint Committee adjudication status), the difference between these groups did not reach statistical significance at 0.025 level. Regarding the secondary outcomes, there was a non-significant reduction in WHF or all-cause death or readmission due to HF up to day 14. Moreover, the serelaxin group demonstrated a significantly smaller number of patients with persistent signs or symptoms of HF at each visit up to day 4 when compared to SoC alone [31].

A meta-analysis was conducted including 6 trials from the serelaxin program (Pre-RELAX-AHF, RELAX-AHF-Japan, RELAX-AHF, RELAX-AHF-2, RELAX-AHF-EU, RELAX-AHF-ASIA) [31]. The results showed that serelaxin had a substantial significant impact on reducing the WHF risk by 23% (RR 0.77, 95% [CI 0.67–0.89] P = 0.0002) and all-cause mortality risk with an HR of 0.87 (95% [CI 0.77–0.98]; P = 0.0261). In contrast, it did not show effect on cardiovascular mortality. Additionally, this analysis showed that serelaxin administration is associated with a significant decrease in the level of markers of renal function and troponin [31].

### Coronary artery disease

Coronary artery disease (CAD) is well-known for its worldwide cardiovascular burden. It represents the most common HF aetiology, as well as the most common comorbidity in HF patients [34, 35]. Prior large clinical trial populations involving HF patients, such as RELAX-AHF and RELAX-AHF-2, revealed a substantial incidence of patients with concurrent CAD [24, 27]. Relaxin-2 pharmacological effects on the vasculature, such as arterial vasodilation and increased arterial compliance, are thought to be mediated via the enhanced production of NO and VEGF, and by the suppressed production of endothelin-1 and angiotensin II [13]. These effects have been investigated in pre-clinical studies demonstrating that relaxin may help to reduce renal and cardiac ischemia–reperfusion (IR) injury [36, 37]. Thus, while serelaxin treatment has the potential to enhance coronary and systemic macrovascular function, leading to improved myocardial perfusion (MP), its vasodilatory effects could also potentially decrease coronary artery perfusion and be deleterious for CAD patients [38].

A recent study investigated the impact of serelaxin on patients with stable CAD, focusing on its effects on both the coronary and systemic vasculature [38]. The study measured MP and aortic stiffness parameters after serelaxin administration and assessed changes in myocardial perfusion reserve (MPR) and Augmentation Index (Alx) from baseline. The results from this study are summarized in Table 1. The study found that a 48 h serelaxin infusion reduced blood pressure, but did not significantly alter MP or aortic stiffness, suggesting that it does not have a significant effect on the microvascular or macrovascular function in established stable CAD patients [38]. The neutral effects on mean global myocardial blood flow (MBF) and MPR is intriguing, particularly when compared to other vasodilators, which are known to cause a reduction on local MP [39, 40]. Thus, this suggests that serelaxin may have a different mechanism of action compared to other vasodilators and may not affect microvascular or macrovascular function in stable CAD in the same way [38]. The study, however, had some methodological issues to discuss: first, a 48-h infusion of relaxin cannot be expected to affect structural vessel parameters such as stiffness, as these changes require extensive remodeling. Indeed, previous pre-clinical investigations that have shown improvements in vascular function and anti-fibrotic properties have used prolonged treatment protocols. Second, and with regard to coronary microvascular perfusion, it is difficult to interpret stress MRI results in the light of relaxin’s global vasodilating effects and in the presence of obstructive CAD [38]. Additionally, there was a significant decrease in endothelin-1 and cystatin C levels following serelaxin treatment and no clinically relevant changes in the levels of N-terminal pro-brain natriuretic peptide (NT-proBNP) or high-sensitivity *cardiac* troponin T (hsTnTS) [38].

## Discussion

Serelaxin has been considered to hold therapeutic potential in cardiovascular health, particularly in AHF. Although well-tolerated and associated with interesting outcomes, clinical trials have shown inconsistent and inconclusive findings.

The Pre-RELAX-AHF study suggested that early serelaxin administration significantly reduced HF symptoms and cardiovascular mortality. Based on the previously shown vasodilatory effects of relaxin and on the pathophysiology of AHF [41], it was hypothesized that patients presenting higher BP would show the greatest benefit from this therapy, thus, only patients with BP > 125 mmHg were included [21]. This criterion may be a limitation as it targets patients that have a lower risk of in-hospital and post-discharge mortality in comparison with patients with lower BP [42], limiting the generalizability of the treatment for this group of patients [21]. Nevertheless, targeting patients with normal-to-raised BP is noteworthy as it represents the largest subgroup of patients with decompensated HF [35, 42]. Additionally, patients in this study were promptly randomized after presentation with a median time to randomization of 6.6 h, which enhances the validity of this study data, as symptoms tend to improve through time and with SoC therapy. The RELAX-AHF trial results showed that serelaxin-treated patients had greater dyspnea relief primarily due to a reduction in WHF, as well as significant 37% reduction in cardiovascular and all-cause deaths [24]. Despite the favourable results, this trial was not prospectively designed as a mortality trial, which represents a limitation to this findings’ validity [24]. Additionally, patients with lower BP were also excluded from this trial and this must be taken into consideration when assessing mortality outcomes as previously discussed. Moreover, a study found that patients enrolled in the RELAX-AHF trial were largely unrepresentative of global AHF patients, as it only represented 20% of AHF patients in the United States, Latin America or Asian-Pacific region. As a result, the study concluded that the enrolled patients differ from other hospitalized patients in terms of distinct clinical features and outcomes [43]. Significant clinical differences were also detected between enrolled patients in RELAX-AHF in comparison to the Epidemiology of Acute Heart Failure in Emergency Departments (EAHFE) registry [44]. Therefore, subsequent literature warns of the caution of extrapolating these findings to all patients with AHF as the trial population was substantially unrepresentative of global AHF patients [45].

The RELAX-AHF-2 trial was five times larger than the previous RELAX-AHF trial [28]. In this study, serelaxin administration did not result in decreased cardiovascular mortality or WHF in AHF patients [27]. The results in this trial are incongruent with the previous findings in serelaxin trials and explanations as to why this happened are still not clear. Discrepancies between the two studies could have been due to the heterogeneity between participants and their risk profiles, as well as the heterogeneity of HF phenotypes [18, 31].

The challenges in enrolling a sufficient number of AHF patients for clinical trials were highlighted by the RELAX-II study. The use of higher baseline natriuretic peptide levels and lower renal function measures as entry criteria in RELAX-AHF-2 did not result in higher placebo event rates, as would have been expected. In fact, the placebo event rates were lower in RELAX-AHF-2 than in the previous RELAX-AHF study, particularly for all-cause mortality and WHF. This suggests that patients enrolled in RELAX-AHF-2 may have milder disease and thus were potentially less responsive to serelaxin therapy [27]. This finding has important implications for the design of future clinical trials in AHF patients, as the selection of appropriate patients may increase the chances of detecting the real efficacy of new treatments. Indeed, ‘mega-trials’ may be vulnerable to both reductions in placebo event rates and dilutions of the treatment effect, most pronouncedly with disease-specific events such as AHF. The inappropriate heterogeneity of the target population can reduce both placebo event rates and treatment effects and have an adverse effect on the power to detect a real benefit of a new intervention [46].

The inconsistency of findings across the trials may also have been due to the subjective assessment of the primary endpoint—WHF. Unlike the others, RELAX-AHF-EUROPE evaluated adjudicated WHF events by a team of cardiologists. This study showed significant impact only on adjudicated events and not on investigator-reported events, emphasizing the impact of WHF assessment variability [33]. Moreover, as this trial investigated the appearance of WHF by a team of cardiologists, it resulted in a lower WHF incidence compared to the WHF reported by physicians (about one-third of investigator-reported events were excluded) [33]. A posterior article studied the reproducibility of event adjudication of RELAX-AHF-EU and found a discordance of 55% between the RELAX-AHF-EU adjudication and the re-adjudication [47]. This article suggests that events adjudication may help tackle the subjectivity of the assessment of HF and a pre-specified quality-control process should be included in the design of future trials to ensure adjudications reproducibility [47].

Despite the evidence of efficacy in the relief of symptoms, as well as an association with diminished WHF and all-cause mortality risk [31] the neutral mortality findings in RELAX-AHF-2 resulted in a lack of approval of serelaxin (Reasanz^™^, Novartis Europharm Ltd, Sussex, UK) use in the AHF treatment [48]. Although phase III trials constitute stronger evidence to show efficacy or lack thereof, it is crucial to look critically at these results. The mortality-powered phase 3 trial concluded that serelaxin administration may not be effective in decreasing long-term mortality but the hypothesis that these results may not necessarily be due to the ineffectiveness of the treatment cannot be disregarded [31]. Serelaxin and other therapies currently undergoing trials, may not be able to demonstrate significant results in randomized controlled trials due to variables of trial design, patient selection and treatment protocols [18].

### Serelaxin—limitations

Serelaxin pharmacological effects are yet to be fully understood and the negative results of the serelaxin studies for AHF may indicate that AHF may not be the target for its use [48]. AHF complex pathophysiology is largely characterized by cardiac fibrosis which itself is a chronic condition, and serelaxin acute administration may not be sufficient to reach its anti-fibrotic effect [49]. Recently, trials with intravenous vasodilators therapies have been conducted but none succeeded to show benefits on post-discharge mortality. Among those, the TRUE-AHF trial assessed long-term cardiovascular mortality after a short-term 48 h infusion of Ularitide, (similar intervention as serelaxin) but showing no beneficial sustained effect when compared to placebo [50]. This evidence may suggest that short-term interventions may not have an impact on the long-term outcomes of patients hospitalized for AHF [27]. For this purpose, extending the treatment period beyond 48 h may be crucial to fully evaluate serelaxin efficacy as an AHF treatment. Serelaxin intravenous infusion remains a challenge due to its short half-life (estimated to be 2 h) which represents an early loss of in vivo effect, with the need for repeated doses to achieve effective plasma levels, limiting its use to hospitalized patients [48]. A previous study indicated that repeated doses of serelaxin in CHF patients are safe and well-tolerated and conducting appropriately designed phase 3 trials may be important to investigate the efficacy of serelaxin long-term therapy [51]. However, patient compliance would likely present a challenge, hence, the development of long-lasting relaxin mimetics would be necessary.

### Relaxin—future perspectives

Recently, new long-acting human relaxin analogues (Table 3), such as LY3540378 (a monomeric fusion protein that binds to serum albumin), have been developed to overcome serelaxin shortcomings. It is hypothesized that it can represent a better candidate for CHF treatment through its extended half-life and improved pharmacodynamics profile [52]. Indeed, this long-acting relaxin analogue is currently being tested in a phase 2 trial to investigate its safety and efficacy in HF patients with preserved ejection fraction [53]. We await the results of this trial, as they may provide valuable insights into the potential of relaxin-2 mimetics to improve outcomes for this patient population. SA10SC‐RLX is another long-lasting relaxin compound, also developed to be suitable for chronic administration in patients via subcutaneous daily release. Along with a higher bioavailability and convenient subcutaneous administration, it is suggested that long-lasting mimetics may be the next step to investigate the role of relaxin in cardiovascular health. They may serve as a chronic therapy to help treat both AHF and prevent AHF decompensation, mainly during the few months of post-discharge when the patients are the most vulnerable [54].

**Table 3:** Examples of long-acting human relaxin analogues

Compound Name	Type	Relevant features
LY3540378	Peptide agonist of RXFP1 composed of a human relaxin analogue and a serum albumin-binding VHH domain	- Developed using novel half-life extension technology - Improved diastolic dysfunction in a preclinical model of cardiac hypertrophy [52] - Clinical trial ongoing to assess the efficacy and safety in adults with worsening HFpEF [53]
SA10SC‐RLX	Single chain lipidated peptide agonist of RXFP1	- Mimics relaxin activity in in vitro and in vivo models of acute RXFP1 engagement - Potentially suitable for once daily subcutaneous administration in patients [54]
CGEN25009	Peptide agonist of RXFP1	- Prevents preclinical bleomycin-induced pulmonary fibrosis - Relaxin-like effects are dependent upon RXFP1 expression IPF human lungs [59]
B7-33	Single chain peptide agonist of RXFP1	- Anti-fibrotic effects of RLX have been replicated in preclinical models of MI, chronic allergic airways disease and obstructive nephropathy - The vasodilatory effects of RLX have been reproduced by B7–33 in rats and mice [60]
ML290	Non-peptidic RXFP1 agonist	- Humanized RXFP1 mouse serves as an invaluable tool for testing of ML290 - Suppresses fibrotic phenotypes in human cardiac fibroblasts and hepatic stellate cells, and preclinical liver fibrosis - The higher potency for cGMP accumulation indicates that ML290 may be a direct vasodilator [61]

*RXFP1* relaxin family peptide receptor 1, *HFpEF* heart failure with preserved ejection fraction, *IPF* *idiopathic pulmonary fibrosis*, *RLX* relaxin-2, *MI* myocardial infarction, *cGMP* cyclic GMP

## Conclusions

The serelaxin clinical trials failed to show significance due to several reasons, including patient selection, trial design, and the use of long-term clinical endpoints despite the short serelaxin administration. Indeed, future trials should use new methods for examining composite endpoints, such as the win-ratio used in EMPULSE, which can give a larger role to short-term changes in symptoms rather than medium and long-term outcomes [55]. The primary outcome in many cardiovascular trials is a composite that includes nonfatal and fatal events and the time-to-first event analysis gives equal statistical weighting to each component event. The win-ratio, which takes into account the clinical importance and timing of the outcomes, has been suggested as an alternative approach [55].

In conclusion, these limitations may not accurately reflect the potential efficacy of relaxin and, as a result, may be hiding its true therapeutic potential. Hence, new clinical trials should be designed to accurately represent the pharmacological effect of relaxin in HF patients. This will require a focus on developing new strategies for relaxin administration and conducting further clinical research using long-acting relaxin mimetics.
